# Harnessing DNA polymerase beta defect enhances synthetic lethality and treatment response in gastric cancer cells: implication for immunotherapy

**DOI:** 10.3389/jpps.2025.15360

**Published:** 2026-01-06

**Authors:** Aashirwad Shahi, Shengyuan Zhao, Dawit Kidane

**Affiliations:** 1 Department of Physiology and Biophysics, College of Medicine, Howard University, Washington, DC, United States; 2 Division of Pharmacology and Toxicology, College of Pharmacy, Dell Pediatric Research Institute, The University of Texas at Austin, Austin, TX, United States

**Keywords:** DNA repair, DNA polymerase beta, dRP lyase, DNA replication stress, mitosis catastrophe

## Abstract

Gastric cancer remains a highly prevalent and accounts for a notable proportion of global cancer mortality. Both Intrinsic and exogenous agents can exacerbate reactive oxygen species (ROS) related oxidized DNA base lesions and single stranded DNA breaks (SSBs). Base excision repair (BER) serves as the primary defense mechanism for repairing DNA damage induced by oxidative stress. DNA polymerase beta (Pol β) plays a critical role in BER and non-homologous end joining repair pathways. The Pol β is the first perform gap-filling DNA synthesis by its polymerase activity and then cleave a 5′-deoxyribose-5-phosphate (dRP) moiety via its dRP lyase activity. Furthermore, defect in POLB promotes genetic liability of the cancer cells for different targeted and synthetic lethality-based treatment strategies. In this review, we have provided a potential example to illustrate the mechanistic insight how PARP1 inhibitor (Olaparib) induces replication associated double strand breaks in POLB deficient cells and DNA mediated innate immune signal activation that likely enhances immune based therapy. Based on our previously published data and the current recent findings, POLB status of the patient likely provide genetic indicators to stratify gastric cancer patient. Overall, in this review article, we presented a new direction to highlight the opportunity to exploit POLB genetic defect in cancer cells to enhance treatment response and to explore synergistic effect to target gastric cancer cells that harbor aberrant DNA polymerase beta function with immune based therapeutic strategy.

## Introduction

DNA is vulnerable to different environmental agents such as ultraviolet light, ionizing radiation (IR), chemicals, toxins and pollutants [1–3], as well as to endogenously generated alkylating agents and reactive oxygen species [4]. The major source of endogenous DNA damage is reactive oxygen species (ROS) generated from normal cellular metabolism [5]. ROS induces different forms of DNA damage, including oxidized DNA bases, abasic sites, SSBs and DSBs [6]. To avoid the deleterious consequences of DNA damage accumulation, cells employ DNA repair mechanisms to fix damaged DNA [7]. Multiple DNA repair pathways have evolved, each associated with repairing specific types of lesions [8, 9]. Oxidized DNA base lesions and single stranded DNA breaks (SSBs) are primarily repaired by the base excision repair (BER) pathway [10]. BER plays a critical role in repairing up to 20,000 endogenous DNA lesions per cell per day [11–13]. The two main BER pathways are short-patch (SP) or long-patch (LP) repair, which occur following completion of DNA end-processing [14, 15]. The canonical short-patch pathway of BER proceeds by filling of the single-nucleotide gap with sequential removal of 5′-deoxyribose phosphate by 5′-deoxyribose phosphate (dRP) lyase activity and incorporation of nucleotide via the nucleotidyl transferase activity of DNA polymerase β (POLB). Finally, the single-strand break is sealed by the activity of DNA ligase IIIα (LigIIIα) in complex with XRCC1. However, LP-BER usually involves strand displacement synthesis with insertion of up to 10 nucleotides in length. While there is evidence that Pol β can function in LP-BER [16, 17].

Several other studies including our work have shown that BER is essential for preventing gastric cancer induced by spontaneous or exogenous risk factors [18]. However, numerous genetic germline variants and somatic mutations of genes involved in BER significantly modulate the risk of cancer and treatment response [19–21]. The following section of the manuscript provide the substantial evidence regarding the structure and function of POLB to maintain genomic integrity of the cells.

## POLB structure and function

POLB is one of Type-X family of mammalian DNA polymerase and composed of two specialized domains. The smaller 8 kDa N-terminal domain contains the 5′-deoxyribose phosphate (dRP) lyase activity, and the 31 kDa C-terminal domain contains the polymerase activity responsible for DNA synthesis. The 31 kDa polymerase domain is composed of fingers, palm, and thumb subdomains, which are arranged to form *c*atalytic, DNA binding, and N subdomains (*n*ascent base pair binding) reflecting their intrinsic functional roles [22]. The catalytic subdomain coordinates two divalent metal cations that assist the nucleotidyl transferase reaction, whereas other subdomains have critical roles in binding to duplex DNA and nascent base pair (dNTP and templating nucleotide) and are spatially situated on opposite sides of the catalytic subdomain. The DNA polymerase structures that bound to DNA including the incoming complementary dNTP reveals that the N subdomain repositions itself to “sandwich” the nascent base pair between the growing DNA terminus and the polymerase [23]. These enzymatic functions represent two essential steps during the base excision repair (BER) pathway, which is responsible for excision and replacing incorrect as well as damaged bases generated from endogenous as well exogenous DNA damaging agents [24, 25].

## Aberrant POLB is associated with genomic instability and cancer

DNA polymerase beta (POLB) is one of the key player in BER and participates in short and long-patch repair BER pathways [26]. Previous studies demonstrated that POLB is mutated in 40% of human tumors and likely contribute to tumorigenesis through genomic instability [21, 27]. Additionally, *in vitro* and *in vivo* studies have shown that mutation in the polymerase domain of POLB or complete deletion of the POL B gene causes genomic instability in mitotic and meiotic cells, respectively [28, 29]. Eight percent of POLB mutations occur in the dRP lyase domain [21, 30]. The dRP lyase function of POLB plays a major significant role in removal of the 5′-dRP group than any other DNA polymerase [31, 32] and protects cells from DNA damage-induced cytotoxicity [33]. POLB is the primary enzyme responsible for processing the 1-nt gap intermediate in chromatin during SSBR and BER. Single-strand breaks (SSBs) are one of the most prevalent forms of genomic DNA damage, occurring tens of thousands of times per cell per day [12]. If left unrepaired, SSBs can result in mutagenesis, genome instability, and/or cell death [34–36]. To protect the genome, the cell possesses robust single-strand break repair (SSBR) pathways to identify, process, and repair SSBs that arise from various endogenous and exogenous sources [37]. One common mechanism for the generation of SSBs is through decomposition of the sugar-phosphate backbone via oxidation [38], which requires subsequent DNA end processing prior to being repaired directly by the SSBR pathway [39]. SSBs can also form indirectly via the base excision repair (BER) pathway, which is responsible for repairing oxidative and alkylative base damage [38]. These indirect SSBs arise through the enzymatic activity of bifunctional DNA glycosylases or the combined action of a monofunctional DNA glycosylase and AP-endonuclease I (APE1) that creates a 3ʹ-hydroxyl and 5ʹ-deoxyribosephosphate (dRP) nick termini. This substrate is subsequently processed by the AP-lyase activity of POLB creating a 1-nt gap [40, 41]. However, several studies also shows that mutation in the coding region of POLB affect their structure and activity. For instance, mutation in the Polymerase domain of POLB such as E295K, R137Q does not possess any polymerase activity or low polymerase activity respectively [42–45]. Additionally, mutation in POLB at T304I results in the loss of interactive partner such XRCC1 to handle the DNA damage repair suggesting that it will not be scaffolded properly during BER, likely leading to a deficiency in gap filling [46]. Overall, the mutation within the polymerase domain impacts the overall polymerase activity due to the altered DNA substrate binding or positioning of the incoming dNTP or delayed release of the DNA after binding, consequently leading to cellular phenotypes.

## Exploiting POLB for cancer therapy

Clinical studies have also been carried out to investigate the association between aberrant BER and cancer. According to the Cancer Genome Atlas (TCGA) database, mutations of BER genes are common in various cancer types. POLB has been found to harbor mutations in up to 30%–40% of human tumors [21]. Previous studies have shown a correlation between single nucleotide polymorphisms (SNPs) of the POLB gene and the risk to develop various cancers [21, 47]. To increase efficiency and lower the burden of undesirable effects, a major change in cancer therapy is a potential transition from a ‘one-drug-fits-all’ to an individualized treatment approach tailored to the tumor-specific molecular features. For the last 2 decades, there are two main targeted therapeutic strategies proposed and are currently utilized in cancer treatment, both exploiting cancer-specific genetic and metabolic vulnerabilities. In the first approach, therapeutic suppression of aberrantly upregulated oncogenes alleviates the growth advantage of cancer cells. The second approach is based on the phenomenon that genetic alterations acquired by tumor cells cause their dependency on other compensatory pathways, loss of which leads to synthetic lethality. Therefore, therapeutic inhibition of pathways that are synthetic lethal with a cancer-specific alteration evokes cellular death in tumor cells while leaving normal cells unharmed [48]. The recent advent of genome-wide genetic interaction studies has demonstrated the extensive number of synthetic lethal interactions in cancer, many of which can potentially be translated to targeted cancer therapies [49].

## Synthetic lethality in POLB variant in cancer

**FIGURE 1:**
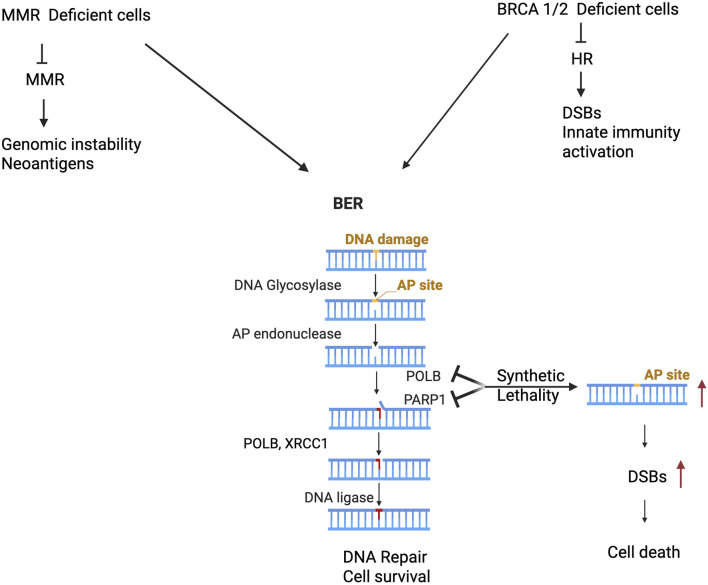
Aberrant POLB induces genetic lability and synthetic lethal with MMR and HR deficient cancer cells. Cancer cells harbor MMR deficient cancer cells leads to accumulation of mutation associated genomic instability, neoantigen generation that potentially generate opportunity for targeting POLB leads to synthetic lethal and potential for immune based therapy (Left panel). Furthermore, other studies provided substantial experimental evidence that targeting POLB in HR deficient cancer (such as BRCA1/2) leads to double strand break associated genomic instability, activation of innate immune signaling and cancer cell death (right panel).

Previous studies have uncovered the significance of synthetic lethality interactions between parallel DNA repair pathways in clinical settings [50, 51]. Targeting PARP1 [Poly(ADP-Ribose) Polymerase 1] for synthetic lethality in preclinical model and clinical setting is therapeutic strategy and approved for patient treatment for several types of cancer. However, not all patients respond due to intrinsic or acquired resistance to PARP1 inhibitor. Dysregulation of POLB is likely a potential genetic liability for cancer cells to enhance sensitivity for treatment. We have examined the following two interactive scenarios across distinct pathways. The first scenario describes the impact of POLB dysregulation and MMR deficiency on cancer cells survival (Figure 1). Some studies have shown that mismatch repair deficient acute lymphoblastic leukemia cells more dependent on POLB-mediated BER pathway to counteract the cytotoxic effects of thiopurine [52]. Additionally, treatment of MMR-deficient cells with APE1 inhibitor in combination with thioguanine caused the accumulation of BER intermediates such as AP sites providing mechanistic insight BER’s role in repairing thioguanine induced DNA lesions in the absence of MMR [53]. Overall, this data suggests a potential therapeutic strategy of targeting BER key factor POLB against MMR-deficient ALL based on synthetic lethality [54, 55].

The second scenario is based on the genetic liability of POLB in HR deficient cancer cells (Figure 1). An estimated 7%–12% of gastric cancers exhibit a mutational signature associated with homologous recombination (HR) failure [56], suggesting that these patients could potentially benefit from PARP inhibitor. Pol β as a synthetically lethal target within BRCA1-deficient cells and a potentially useful one for treating cancer [57]. Additionally, recent study has shown that CRISPR-based target discovery screening identified DNA polymerase beta (POLB) as a synergistic enhancer of the synthetic lethality between PARP and BRCA1/2, supporting *POLB* as a promising therapeutic target for improving antitumor responses to PARP inhibitors in homologous recombination–deficient cancers [58, 59]. Moreover, the following section of this manuscript provides two alternative therapeutic strategy to target the genetic vulnerability of dRP lyase deficient cancer.

## Targeting PARP1 in dRP lyase deficient gastric cancer cells enhance sensitivity

Development of alternative synthetic lethality approaches is a high priority. DNA polymerase β (Polβ), a critical player in base excision repair (BER), interacts with PARP1 during DNA repair. The 5′-dRP activity of POLB is critical for the formation of repair products since the removal of the 5′-dRP group is considered to be a rate-limiting step in short patch BER and is required for ligation of the BER intermediates after gap-filling by a polymerase [60, 61]. Deficiency of 5′-dRP lyase activity of POLB, or the entire POLB gene, leads to increased sensitivity to DNA-damaging agents [33], genetic instability [62], and neonatal lethality [63] respectively. Given the high number of somatic mutations identified in *POLB* gene, we studied L22P mutation in carcinogenesis using *in vitro* and genetic tools. L22P was discovered as a gastric cancer-associated variant of POLB [64]. The L22P variant maps to the 8 kDa lyase domain, which is responsible for the removal of the 5′dRP group. Previous finding showed that the L22P variant retains DNA polymerase activity, but lacks dRP lyase and has less DNA-binding affinity *in vitro* [65]. As a result, L22P is unable to support BER activity. Our *in-vitro* cell lines-based data have shown that in the absence of dRP lyase activity of POLB, BER intermediates trigger a rapid block in DNA synthesis and exert genotoxic effects toward gastric epithelium [66]. L22P leaves the DNA nicks due to its low dRP lyase activity and slower polymerase activity, then these nicks could accumulate in gastric epithelium and be a driver for gastric cancer. Previously, we investigated the biological significance of dRP lyase deficiency result in DNA replication associated genomic instability [66]. Further, we demonstrated that deficiency in dRP lyase in mouse model cause oxidative induced genetic lesions that likely predisposes to gastric cancer [67]. On the other hand, BER intermediates could be processed by alternative DNA repair pathways including non-homologous end joining (NHEJ) [68] or homologous recombination [68]. These alternative DNA repair pathways can induce chromosomal aberrations, eventually resulting in cellular transformation [66]. Taken together, successful completion of BER requires both gap-filling synthesis and 5′-dRP excision by POLB. The genetic liability of cancer cells with dRP lyase deficiency likely provide the opportunity for precision therapeutic strategy and help also to stratify the patients.

**FIGURE 2:**
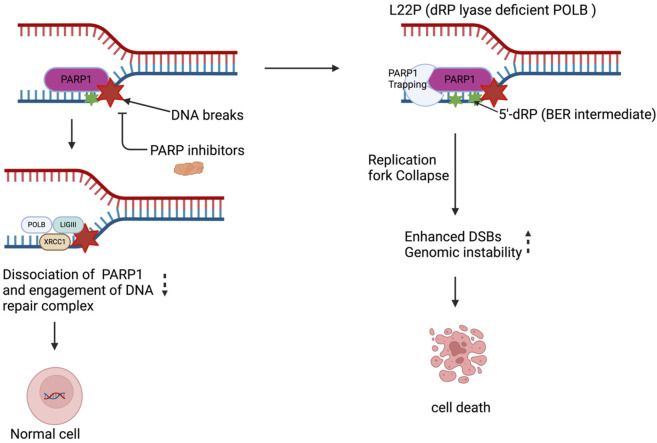
PARP1 inhibitor enhances replication dependent DSBs and sensitizes dRP lyase deficient cells. Targeting PARP1 enhances DNA damage in gastric cancer cells leading to significant genotoxic effects. The L22P mutation generates a 5′-dRP group, which interferes with replication by trapping PARP1. This results in the collapse and stalling of replication forks, ultimately causing an increase in double-strand breaks (DSBs) by inhibiting BER pathway. These DSBs exacerbate genomic instability that leads to cell death.

PARP1 is one of the most abundant and active PARP, strongly activated in response to DNA damage. It synthesizes majority of PAR in response to genotoxic or oxidative stress [69, 70]. PARP1 involved as a DNA nick-sensor and interacts with BER intermediates including 5′dRP groups to facilitate the repair process. Under normal physiological conditions (normal cells), when PARP1 binds to DNA breaks, PARP1 catalyzes synthesis of poly (ADP-ribose) covalently attached to itself and some nuclear proteins and auto poly ADP-ribosylation of PARP1 facilitates its dissociation from DNA breaks and is considered as a factor regulating DNA repair. However, in cancer cells with harboring mutation that able accumulate 5′dRP groups likely provide the opportunity of PARP1 to stay longer on the DNA substrate and causes genotoxicity. Our previously published work shows that L22P expressing cells exhibit hypersensitivity to PARP inhibitor treatment due to an accumulation of double-strand breaks. The mechanism for increased PARP inhibitor sensitivity is due to PARP1 interacting with the 5′dRP groups that L22P Pol β fails to remove and becomes trapped on the DNA causing replication fork stalling and DSB break formation [66]. Replication forks that have been stalled by trapped PARP1 collapse during S-phase results in DSBs [71] suggesting that L22P-expressing cells accumulate 5′-dRP groups, which are critical for interaction with PARP1. Our study confirmed that treatment with a PARP1 inhibitor eliminates dRP lyase deficient cells via trapping a PARP1- 5′-dRP group complex which suggests that trapped PARP1 may likely blocked replication forks that ultimately leads to DSBs. Further, there is also evidence the DNA protein crosslink formation with POLB depends on the enzyme’s lyase activity. Our previous experimental observation is in agreement with previous reports that PARP1 forms a covalent bond with 5′-dRP groups and blocks BER or hinders the BER process [72]. Therefore, PARP1 trapping and/or Pol β forming DPCs, likely contribute to the DNA damage accumulation observed in this L22P variant of POLB, demonstrating the importance of the lyase domain for proper POLB function (Figure 2). Therefore, the genetic vulnerability of cancer cells that harbor POLB polymorphism at dRP lyase domain or somatic mutation likely cause the perturbs the BER pathway and sensitizes cancer cells to PARP1 inhibitors. Our observation may imply that gastric cancer patients carrying defects in POLB function may be stratified for PARP1 inhibitor treatment, resulting in a more effective option.

## Targeting PARP1 exacerbate DNA mediated innate immune signaling in dRP lyase deficient gastric cancer

**FIGURE 3:**
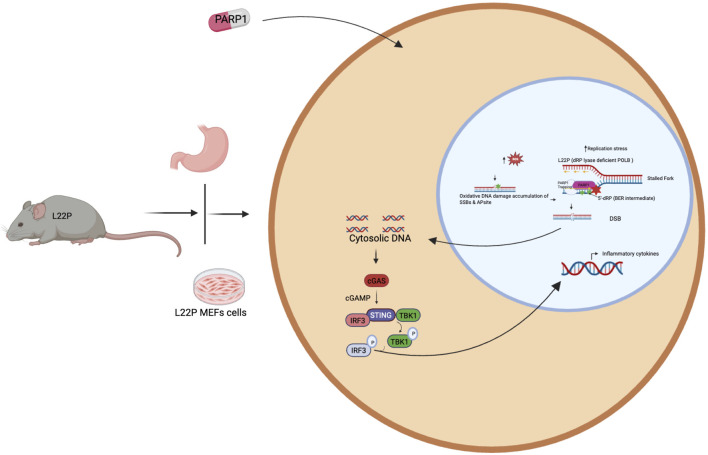
PARP1 inhibitor induces DNA mediated innate immune signaling activation in dRP lyase deficient gastric cancer. Accumulation of unrepaired DNA damage in dRP lyase deficient cells likely generate cytosolic DNA to stimulate cGAS/STING pathways. Further inhibition of PARP1 blocks the replication forks and unresolved repair exacerbate the release of cytosolic DNA that leads to cGAS-STING activation and production of inflammatory cytokines.

Several studies have suggested that DNA repair factors play a role in modulating an inflammatory response [73, 74]. Once nuclear DNA integrity is compromised through a deficient DNA repair system or exogenous DNA damaging agents, cells will likely release the DNA into the cytosolic compartment and possibly activate STING signaling and engage an inflammatory response. Our published work demonstrated that a dRP lyase deficient variant of POLB (Leu22Pro, or L22P) spontaneously induces genomic instability, which eventually leads to a cytosolic nucleic acid mediated inflammatory response [75]. Furthermore, poly(ADP-ribose) polymerase 1(PARP1) inhibition exacerbates chromosomal instability and enhances the cytosolic DNA mediated inflammatory response [75]. It is well documented that chronic stimulation of the immune system is critical for tumor promotion and progression [76, 77]. One of the key interfaces between defective DNA repair and immunogenicity is the cyclic GMP-AMP synthase/stimulator of IFN genes (cGAS/STING) pathway [77]. The cGAS-STING pathway, which senses cytosolic DNA, has been linked to an anti-tumor inflammatory response [78]. Cytosolic double-stranded DNA is sensed by cGAS, leading to activation of the transmembrane protein STING and activation of the transcription factors interferon regulatory factor 3 (mainly IRF3) and nuclear factor kappa B (NF-κB) followed by an upregulation of interferon beta (IFN-β) related genes [79, 80]. Loss of dRP lyase in mice stomach provided an excellent model to demonstrates that the interplay between aberrant BER and inflammation in gastric cancer [81]. Our recent work have shown that dRP lyase deficient POLB triggers cytosolic DNA mediated chronic inflammation in L22P mice STING has recently been identified as one of the critical adaptors for sensing cytosolic DNA, followed by the phosphorylation of IRF3 and subsequent production of type-I IFN and IL-6 [75]. Furthermore, the mRNA expression of interferon type-I cytokines significantly increased in the stomach tissues of dRP lyase deficient mice [75]. Our study reveals a previously unidentified role of POLB in regulating the cellular inflammatory response thus providing a potential target in a defective BER pathway to enhance an immune based therapy response in the future (Figure 3).

## BER deficiency generate therapeutic opportunity for immune check point blocked

**FIGURE 4:**
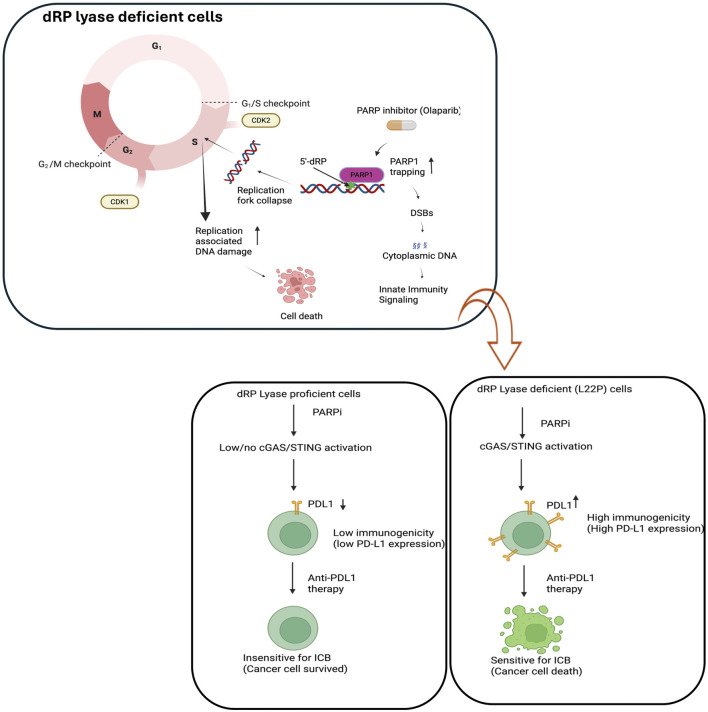
Potential impact of dysregulated BER because of dRP lyase deficiency may enhance sensitivity to immune checkpoint blockade (ICB). The model demonstrates the combinatorial effect of PARP inhibitor (Olaparib) and exploiting replication stress and DNA damage to enhance immunogenicity. Replication fork collapse during the S phase leads to DNA damage, requiring PARP1-mediated repair via base excision repair (BER). PARP1 inhibitors impair BER resulting in accumulation of DNA damage and activation of cGAS/STING. This model likely implies the upregulation of PD-L1 may open the opportunity to be targeted by anti-PDL1 antibody to effectively treat the patients with dRP lyase deficient cancer. Figure created with BioRender.com.

The tumor microenvironment composition including CD8 + T, CD4 + T, macrophages and tumor-associated fibroblasts relates with clinical outcomes in various cancers including gastric cancer [82]. Other studies have shown that DNA repair deficiency has been shown to be associated with immunogenicity in other types of cancer [83]. One such therapy in particular, that is, anti-programmed cell death protein 1 (PD-1)/programmed death-ligand 1 (PD-L1) antibody therapy has been widely applied to treat several types of cancer [84, 85]. Anti-PD-1/PD-L1 antibody restores antitumor immune responses by disrupting the interactions between PD-1 and its ligand, PD-L1, thereby providing effective antitumor effects by augmenting the body’s own immune activity against the tumor. However, patient responses to this treatment are highly variable; anti-PD-1/PD-L1 antibodies alone produce dramatic response rates for high responders (∼5% of patients), whereas approximately 40% of patients show cancer progression despite treatment [86–88]. Experimental data suggests that cancer cells with multiple gene mutations (high mutational loads) show higher PD-L1 expression in tumor tissue [89]. In line with this data, other studies also support that high immune therapy response rates of cancers with microsatellite instability (MSI to anti-PD-1 therapy have been reported [90, 91]. Previous studies have shown that tumor infiltration by immune cells is linked with prognosis of gastric cancer patients [92]. Furthermore, several studies demonstrated that gastric cancer and other types of tumors harbor significant BER defects [20, 21, 93, 94]. Exploiting those BER defect factors enhances program cell death ligand-1 (PD-L1) expression in cancer cells. Overwhelming evidence have shown that the PD-L1 level of expression in tumors is an important factor to influence the therapeutic efficacy of response of cancer patients [95, 96]. Furthermore, emerging evidence suggests that defects in DNA repair machinery led to upregulation of PD-L1 [97]. Permata et al. also showed that BER gene expressions are negatively correlated with PD-L1 expression in tumors and oxidative DNA damaging agents exacerbate the expression of PD-L1 [98]. In line with this our recent publication showed that BER defects/low expression show high microsatellite instability increased neoantigen production and PD-L1 expression in tumors [99]. In addition, chemotherapeutic agents inducing DNA damage enhanced the expression of PD-L1 in many cancer types [100]. Furthermore, resistance to PD-1/PD-L1 inhibitors, whether primary or acquired, remains a significant challenge [101]. Therefore, there is an urgent need for new therapeutic strategies in clinical practice to further increase the efficacy of immunotherapy. Considering this issue, our review provides that dysregulated BER function likely engages the tumor immune microenvironment as a novel strategy to increase the sensitivity of GC to ICIs to use ant-PDL1 or anti-CTL4 antibody. Exploring BER deficiency and oxidative stress DNA damage associated upregulation of PD-L1expression in tumors will likely provide an additional immune biomarker to introduce the immune-based therapeutic strategy (Figure 4).

## Discussion

Cancer cells frequently acquire mutations in DNA repair genes and respond by rewiring their DNA repair network to utilize compensatory pathways for survival. Dependency on compensatory DNA repair pathways opens room for the development of cancer-specific small molecule inhibitors. Persistent DNA damage can be genotoxic or cytotoxic for the cells. POLB is required to maintain genomic stability, however mutation associated with loss of function is a genetic lability of the cells and provide opportunity to enhance treatment response. Additionally, POLB could be a potential target for cancer cells that are deficient MMR and BRCA1/2 to promotes synthetic lethality. In this regard, we provided example that have focused on PARP’s role in the base excision repair (BER) pathway, to gauges the extent of damage and functions as a scaffold or stabilizer for dRP lyase deficient cells. We found that dRP lyase deficient cancer cells is significantly more sensitive towards Olaparib potentially due to DNA replication block at S-phase cell cycle stage (Figure 2). Overall, our study provides experimental evidence that PARP inhibition abrogates BER functionality, causing accumulation of unresolved single-stranded breaks (SSBs) that convert to double-stranded breaks (DSBs) during S phase and promotes cancer cell death. This result suggests that deficiency or mutations in dRP domain of POLB represent a potential vulnerability in cancer cells, offering alternative molecular target for selecting and optimizing precision therapeutic strategy for patients with aberrant BER. In addition, targeting the PARP1 likely stimulate innate immune signaling to promote immune based therapy in gastric cancer (Figures 3, 4). The advent of anti-PD-1/anti-PD-L1 has transformed the therapeutic landscape for GC, introducing a novel avenue for harnessing the immune system against tumor cells. The future may uncover potential mechanistic insight into how the DNA repair pathway modulates the innate immune response in terms of reprogramming the tumor microenvironment, restoring antitumor immunities, and enhancing cancer immunotherapy treatment response.
